# Inbred Mouse Populations Exhibit Intergenerational Changes in Intestinal Microbiota Composition and Function Following Introduction to a Facility

**DOI:** 10.3389/fmicb.2017.00608

**Published:** 2017-04-11

**Authors:** Jocelyn M. Choo, Paul J. Trim, Lex E. X. Leong, Guy C. J. Abell, Carly Brune, Nicole Jeffries, Steve Wesselingh, T. N. Dear, Marten F. Snel, Geraint B. Rogers

**Affiliations:** ^1^Infection and Immunity Theme, South Australian Health and Medical Research Institute, AdelaideSA, Australia; ^2^Lysosomal Diseases Research Unit, Nutrition and Metabolism Theme, South Australia Health and Medical Research Institute, AdelaideSA, Australia; ^3^School of Medicine, Flinders University, AdelaideSA, Australia; ^4^Bioresources facility, South Australia Health and Medical Research Institute, AdelaideSA, Australia

**Keywords:** C57BL/6J inbred mice, mice generations, fecal microbiota, fecal metabolome, microbiome variation

## Abstract

Inbred mice are used to investigate many aspects of human physiology, including susceptibility to disease and response to therapies. Despite increasing evidence that the composition and function of the murine intestinal microbiota can substantially influence a broad range of experimental outcomes, relatively little is known about microbiome dynamics within experimental mouse populations. We investigated changes in the intestinal microbiome between C57BL/6J mice spanning six generations (assessed at generations 1, 2, 3, and 6), following their introduction to a stringently controlled facility. Fecal microbiota composition and function were assessed by 16S rRNA gene amplicon sequencing and liquid chromatography mass spectrometry, respectively. Significant divergence of the intestinal microbiota between founder and second generation mice, as well as continuing inter-generational variance, was observed. Bacterial taxa whose relative abundance changed significantly through time included *Akkermansia, Turicibacter*, and *Bifidobacterium* (*p* < 0.05), all of which are recognized as having the potential to substantially influence host physiology. Shifts in microbiota composition were mirrored by corresponding differences in the fecal metabolome (*r* = 0.57, *p* = 0.0001), with notable differences in levels of tryptophan pathway metabolites and amino acids, including glutamine, glutamate and aspartate. We related the magnitude of changes in the intestinal microbiota and metabolome characteristics during acclimation to those observed between populations housed in separate facilities, which differed in regards to husbandry, barrier conditions and dietary intake. The microbiome variance reported here has implications for experimental reproducibility, and as a consequence, experimental design and the interpretation of research outcomes across wide range of contexts.

## Introduction

Murine models have contributed substantially to biomedical research, from investigations of basic physiology to assessments of therapies and clinical procedures (Rabadan-Diehl and Nathanielsz, 2013). The use of mice not only allows experiments that would not be acceptable in humans, but also facilitates the control of variables, such as genetic differences, that could otherwise confound experimental outcomes. The importance of maintaining genetic uniformity in experimental populations is well-recognized (Fahey et al., 2013). However, there is increasing evidence from studies involving both human and mouse that the gut microbiome has a major influence on many aspects of physiology and disease susceptibility. For example, the intestinal microbiome has been shown to be an important regulator of both metabolic homeostasis (Sonnenburg and Backhed, 2016) and adaptive and innate immunity (Jarchum and Pamer, 2011). There is even compelling evidence that host-microbe interactions directly affect brain development and cognition (Rogers et al., 2016). Disruption of these homeostatic roles through perturbation of the normal microbial balance is associated with an increasing number of pathological conditions; including obesity, autoimmune diseases, chronic gastrointestinal inflammatory diseases, type II diabetes, depression, and cancer (Carding et al., 2015). In addition, the influence of the intestinal microbiota in the efficacy of therapies is reflected through their roles in the reductive metabolism of pharmaceuticals (Liang et al., 2015) and the production of hepatic enzymes involved in systemic drug metabolism (Meinl et al., 2009).

An appreciation of the potential for variation at the microbiome level to influence the outcomes of animal-based experiments is growing. A recent high-profile study showed that differences in melanoma growth were linked to the differences in intestinal microbiota composition of mice obtained from separate commercial suppliers (Sivan et al., 2015), while differences in microbiota composition have also been implicated in susceptibility to both dextran sodium sulfate-induced (Brinkman et al., 2013) and *Helicobacter hepaticus*-induced colitis (Yang et al., 2013).

Such microbiota-level differences can arise due to internal or external influences on the gut environment. For example, genetic background (Deloris Alexander et al., 2006; Campbell et al., 2012), diet, age (Langille et al., 2014; Ericsson et al., 2015) and to a lesser extent, sex (Kovacs et al., 2011; Campbell et al., 2012) have all been shown to influence the intestinal microbiota of mice. Differences can also arise through stochastic processes. For example, the physical separation of animals facilitates microbiota drift, a process that has been demonstrated both within (Campbell et al., 2012; McCafferty et al., 2013; Rogers et al., 2014; Hoy et al., 2015; Jakobsson et al., 2015) and between (Friswell et al., 2010; Rausch et al., 2016) animal facilities. Separation of knockout (KO) and control strains (Ubeda et al., 2012) have been demonstrated to contribute to the phenotypic characteristics reported for KO. Together, these factors are likely to contribute to the substantial differences in intestinal microbiota in mice obtained from different commercial suppliers (Friswell et al., 2010; Hufeldt et al., 2010; Ericsson et al., 2015). Importantly, differences in microbiota composition can have a profound influence on the metabolites and excreted factors that the commensal intestinal bacteria collectively produces (Claus et al., 2008; Wikoff et al., 2009; Rogers et al., 2014). Referred to as the intestinal metabolome, this extracellular milieu is a key mediator of many microbiota-host signaling interactions, and influence disease and health outcomes (Andoh et al., 2003; Vinolo et al., 2011). The microbial community of mice were previously observed to undergo a period of equilibration and stabilization when transferred from a mouse farm or vendor into an animal facility (Fushuku and Fukuda, 2008b; Hoy et al., 2015). However, the extent to which these changes dissipate or persist over successive generations is not known.

The vertical transmission of microbes between dams and pups has been shown to be important in influencing intestinal microbiota composition (Friswell et al., 2010; Daft et al., 2015). The reconstitution of the maternal microbiota in offspring is an imperfect process that is likely to contribute substantially to bacterial dispersal within a population (Rausch et al., 2016; Sonnenburg et al., 2016). Our study sought to investigate microbiome-level inter-generational differences within a population following its introduction to a controlled animal facility. Specifically, we hypothesized that significant differences in intestinal microbiota composition and function would exist at a population level both in the generation immediately following introduction, and between subsequent generations of mice within the same lineage. Further, we hypothesized that these differences in bacterial taxa and fecal metabolites would have the potential to significantly influence host physiology. Accordingly, we assessed fecal microbiota composition and functional output in inbred C57BL/6J mice across six generations following their introduction into a strictly regulated animal facility.

## Materials and Methods

### Mouse Populations

Mouse studies were conducted according to the institutional animal ethics committee guidelines (SAHMRI Animal Ethics Committee, Adelaide, SA, Australia). Mice involved in the inter-generational study were bred from the C57BL/6J strain. “Generation” refers to the generation post-arrival at the facility, with the founder mice (G1) originally obtained from the Jackson Laboratory (Bar Harbor, ME, USA). G1 were housed and subsequently bred at the South Australian Health and Medical Research Institute Bioresources Facility (Adelaide, SA, Australia) to obtain G2, G3, and G6 mice. A schematic diagram for the breeding of mice generations is detailed in Supplementary Figure S1.

Mice were weaned at 3–4 weeks of age, distributed to individually ventilated cages within a single holding room, fed an identical diet (Teklad Global 18% Rodent Protein Diet, Harlan Laboratories, USA), maintained under the Federation of European Laboratory Animal Science Associations (FELASA) standards and routinely screened using a SNP genotyping panel. Fecal samples were collected from 6 to 24 weeks old mice from the founder generation, (G1, *n* = 24), and the second (G2, *n* = 12), third (G3, *n* = 25) and sixth generation (G6, *n* = 22). Breeding scheme is provided in the Supplementary Figure S1.

For the assessment of within-individual variation, fecal samples were collected from mice (*n* = 8) at ages of 4, 8, and 16 weeks old (corresponding to 1, 5, and 13 weeks post-weaning (PW), respectively).

To assess inter-facility variation, mice from the Flinders University School of Medicine (Adelaide, SA, Australia) (referred to as AF2) were compared to G6 mice at the South Australian Health and Medical Research Institute (referred to as AF1). Mice at the AF2 (*n* = 21) were derived from the Animal Resources Centre (Murdoch, WA, Australia), and previously from the Jackson Laboratory. The AF2 mice were maintained under different conditions compared to those from AF1, including the use of conventional caging, a different diet (Rat and mouse premium breeder diet 23% protein, Gordon’s Specialty Stockfeed, Australia) and SPF-conditions. Sex distribution did not differ significantly between study groups.

### DNA Extraction and 16S rRNA Gene Amplicon Sequencing

Fecal samples were collected by placing individual mice in a clean cage. Fresh fecal pellets were transferred using a sterile toothpick to a 1.5 mL Eppendorf tube and stored at -80°C prior to analysis. Fecal pellets were dispersed in 1 mL of phosphate buffered saline (PBS, pH 7.2) by vortexing, and pelleted by centrifugation at 13 000 ×*g* for 5 min. Supernatant was transferred to a sterile 2 mL screwcap tube and stored at -80°C for liquid chromatography mass spectrometry (LC-MS) analysis, while pellets underwent DNA extraction by a combination of mechanical and chemical cell lysis methods using the PowerSoil^®^-htp 96 Well Soil DNA Isolation kit (Mo Bio Laboratories, Carlsbad, CA, USA). Amplicons of the V4 hypervariable region of the bacterial 16S rRNA gene was amplified from fecal DNA extracts as described previously (Choo et al., 2015). Briefly, modified universal bacterial primer pairs 515F (5′-TCGTCGGCAGCGTCAGATGTGTATAAGAGACAGGTGCCAGCMGCCGCGGTAA-3′) and 806R (5′-GTCTCGTGGGCTCGGAGATGTGTATAAGAGACAGGGACTACHVGGGTWTCTAAT-3′), with Illumina adapter overhang sequences (indicated by underline) were used for the amplification of the V4 hypervariable region of the bacterial 16S rRNA gene. Amplicons were generated from DNA extracts (25 PCR cycles for amplicon generation, followed by eight PCR cycles for indexing), cleaned and sequenced according to the Illumina MiSeq 16S Metagenomic Sequencing Library Preparation protocol with certain modifications. Specifically, PCR for amplicon generation was performed at melting temperature of 50°C. Amplicon sequencing was performed using an Illumina MiSeq at the David R Gunn Genomics Facility, South Australian Health and Medical Research Institute. Full details are provided in Supplementary Material.

### Bioinformatics Analysis

Paired-end 16S rRNA gene sequence reads were analyzed with the Quantitative Insights Into Microbial Ecology (QIIME) software (v1.8.0) (Caporaso et al., 2010) using a bioinformatics pipeline described previously (Jervis-Bardy et al., 2015). Briefly, barcoded forward and reverse sequencing reads were quality filtered and merged using Paired-End reAd mergeR (PEAR v0.9.6) (Zhang et al., 2014). Chimeras were detected and filtered from the paired-end reads using USEARCH (v6.1) (Edgar, 2010) against the 97% clustered representative sequences from the Greengenes database (v13.8) (McDonald et al., 2012). Operational taxonomic units (OTUs) were assigned to the reads using an open reference approach with UCLUST algorithm (v1.2.22q) against the SILVA database release 123 (May 2016) (Quast et al., 2013) that was clustered at 97% identity. During the OTU assignment, sequences were pre-clustered at 80% against the reference prior to *de novo* clustering. All samples were subsampled to 6,175 reads based on the lowest read depth. Goods coverage was 98.5% ± 0.003% (mean ± SD, range: 97.8–99.7%), indicating sufficient resolution of the microbial community at this subsampling depth. Reported taxon relative abundances were inferred based on 16S rRNA gene read abundance. Sequence data is publicly available in the Sequence Read Archive (SRA) repository under the accession number SRP080894.

### LC-MS Analysis

Fecal supernatant, derived from 2.5 mg of fecal matter in PBS, was thawed and vortexed. LC-MS was performed on a quadrupole orthogonal acceleration time-of-flight mass analyzer (SYNAPT HDMS, Waters Corporation, Milford, MA, USA) coupled to a UPLC system (ACQUITY, Waters Corporation, Milford, MA, USA). Reverse-phase chromatographic separation used a Waters BEH C18 column, 1.7 μm particle size, 2.1 mm i.d. × 150 mm (Milford, MA, USA). To assess LC-MS system variability throughout an analysis run, a pooled biological quality control (PBQC) was prepared by combining 10 μL from each sample. Two mass spectrometry experiments were performed, one in negative and one in positive ionization mode. Detailed LC-MS protocols are provided as Supplementary Materials and Methods in Supplementary Material.

### Statistical Analysis

Microbial data were analyzed for alpha diversity measures (taxa richness, Taxa_*S*; taxa diversity, Shannon-Wiener index *H*; taxa evenness, Simpson diversity index, 1-*D*) of microbial community using PAST (v.3.04) (Hammer et al., 2001). OTU relative abundance was imported into the Primer-E software (v.6, PRIMER-E Ltd, Plymouth, UK) for beta diversity analysis. Bray–Curtis similarities were calculated based on the square root-transformed OTU relative abundances, and were used in the non-metric multidimensional scaling (NMDS) ordination plot. Permutational analysis of variance (PERMANOVA) model was used for testing the null hypothesis of no difference between the compared groups (Anderson and Walsh, 2013), based on the permutation of residuals under a reduced model and a type III sum of squares. The bacterial taxa that contributed to significant dissimilarities between groups were determined by SIMPER analysis (Clarke, 1993). PERMDISP was used to assess the dispersion of the microbial community within the groups (Anderson and Walsh, 2013). Comparisons between the microbial and metabolome abundance data was performed using the RELATE analysis (Clarke and Warwick, 2001). Differences in the relative abundance of phyla and genera between generation groups were, where possible, tested for statistical significance based on a nested ANOVA analysis (litters nested in generation), using a type III sum of squares approach on log transformed values in SPSS (v22.0). Multiple pairwise comparisons between groups were performed on the estimated marginal means, with Bonferroni correction applied. Comparisons between generation and within-individual variance was performed using the Kruskal–Wallis test with Dunn’s multiple comparison test using GraphPad PRISM 6 (GraphPad Software Inc., La Jolla, USA). Heatmap was generated in R using the *ggplots2* package (v2.0.0) (R Core Team, 2015; Wickham and Chang, 2015). Unassigned taxa comprised less than 0.1% of the relative abundance and were not included in the phyla relative abundance comparison. The DNA and metabolome samples of two mice from G1 and one mouse from G6 failed quality control thresholds and were excluded from microbiota and metabolome analysis, respectively, as the number of observed taxa were very low (less than 20) and the sample was too dilute. G1 mice, and five G6 mice, were excluded from assessment of litter effects as data for the maternal origins of these mice were not available.

Liquid chromatography mass spectrometry-based metabolomics data were processed using Progenesis QI software (v2.2, Nonlinear Dynamics, Newcastle Upon Tyne, UK). Detailed processing and analysis pipeline for these data are provided as Supplementary Materials and Methods in Supplementary Material. All putative metabolites were assigned based on molecular weight. Statistical analysis were performed at a significance level of 0.05.

## Results

### Variation in Fecal Microbiota Alpha Diversity

**FIGURE 1:**
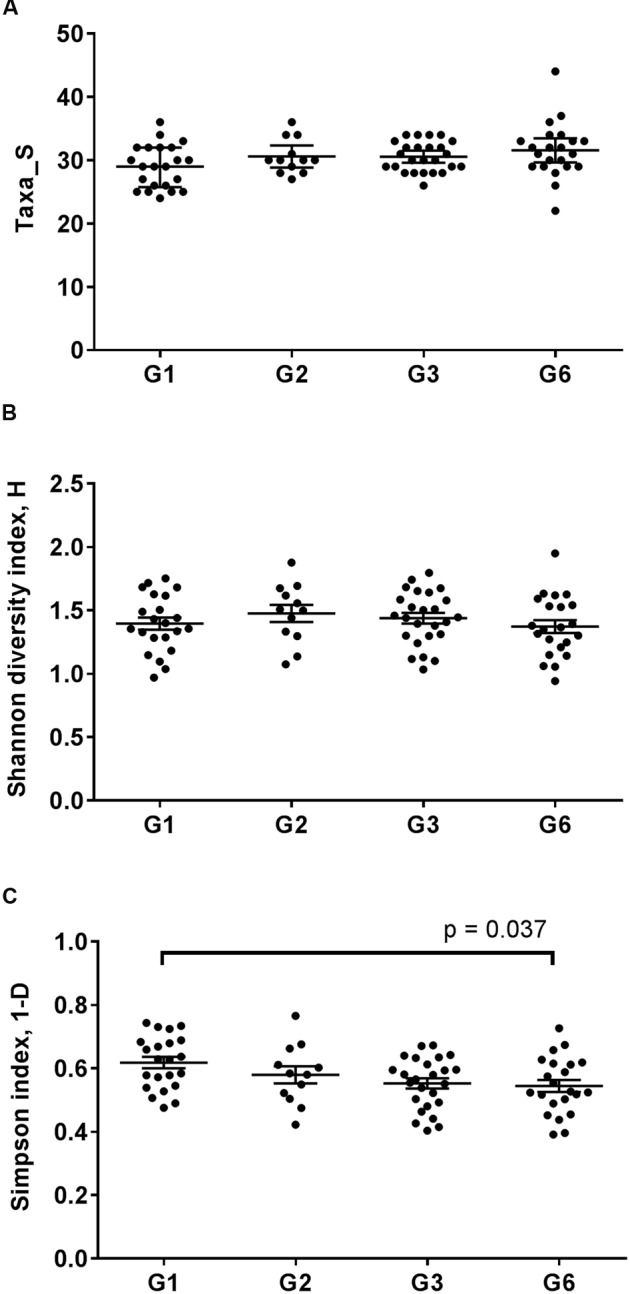
**Alpha diversity analysis of the fecal microbiota of C57BL/6J inbred mice across four different generations.** The fecal microbial diversity of mice were measured based on **(A)** microbial richness **(B)** Shannon-Wiener index *H* for microbial diversity and **(C)** Simpson index 1-D as a measure of microbial evenness, which takes into consideration microbial richness. Each point represents a sample. The middle bar represented the mean and the error bars represented the standard error of mean for each mice generation. The significance of differences between group means was determined using ANOVA with Bonferroni correction for pairwise comparisons.

The microbiome dynamics associated with the introduction of a new mouse population to a facility were assessed by comparing intestinal microbiota structure in the founder stock (G1) with that in the subsequent generation (G2). Further, the extent to which inter-generational differences between populations bred within the same facility persisted, were also assessed (G2 to G6). Mean microbiota evenness (Simpson index) was significantly lower only in G6 (*p* = 0.037) compared to G1 (**Figures 1A–C**). These results suggested notable shifts in microbiota composition that were characterized by an increasing dominance of several taxa in the gut microbiota, which occurred beyond the initial transition from founder population to second generation offspring.

### Variation in Fecal Microbiota Composition

**FIGURE 2:**
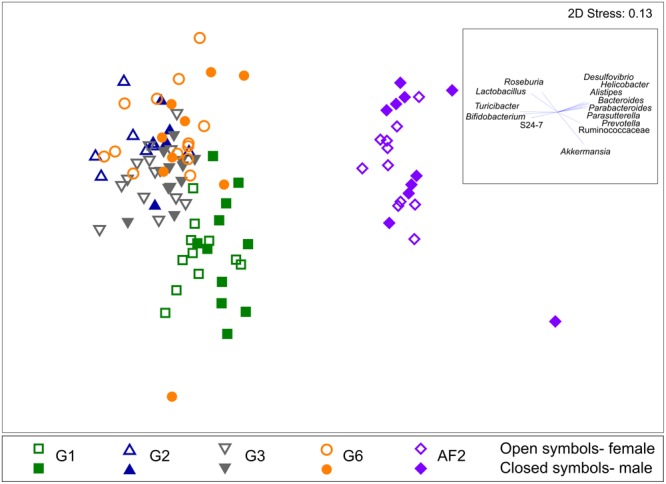
**Non-metric multidimensional scaling (NMDS) ordination plot of the fecal microbiota.** NMDS plot was generated based on the Bray–Curtis distances of the fecal microbiota of C57BL/6J inbred mice from the founder (G1), second (G2), third (G3), and sixth (G6) generation within a single facility, and the same strain of mice from a separate animal facility, AF2. Mice from the different generations and facility and mice sex are labeled as indicated. Bacterial taxa that contributed to the differences between the mice groups are shown as vectors based on a Spearman correlation of >0.4, with the corresponding weight and direction shown in the box inset.

The significance of changes in microbiota composition among generations was assessed by PERMANOVA. Significant differences were identified across the mice generations (PERMANOVA *p* < 0.0001, 9916 permutations; Pseudo-*F* = 12.65, square root ECV: Gen = 12.25, residual = 15.99) and in pairwise analysis of each generation (Supplementary Table S1). Based on t-statistic values, changes in microbiota composition were most substantial between G1 and G2 (*t* = 4.23, Bonferroni-adjusted *p* = 0.0006), with moderate differences between G2, G3, and G6 (*t* = 2.00 to 2.95, Bonferroni-adjusted *p* ≤ 0.003). This pattern can be illustrated by NMDS based on Bray–Curtis distances (**Figure 2**; G1 to G6 only NMDS plot in Supplementary Figure S2; compositional data shown in Supplementary Figure S3), where generational clustering is evident, particularly between G1 and subsequent generations.

**FIGURE 3:**
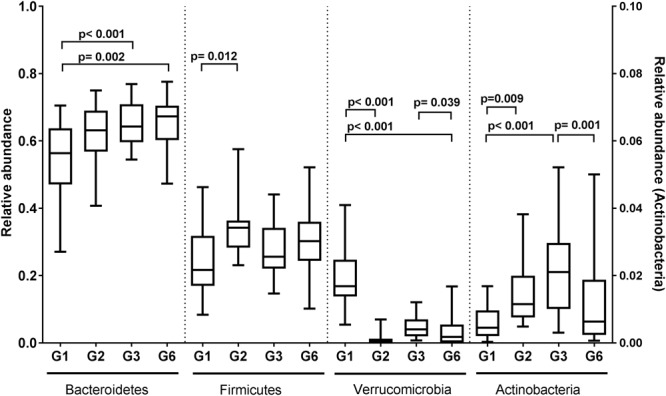
**Relative abundances of the major phyla among C57BL/6J mice generations.** Box-and-whiskers plot of the phyla relative abundances (proportion) in four different generations of C57BL/6J inbred mice. The right *y*-axis was used to plot the relative abundances (proportion) of Actinobacteria. The whiskers bars represented the minimum and maximum points. Statistical significance was determined at a level of *p* < 0.05 using ANOVA with Bonferroni correction.

At the phylum level, significant changes in the mean relative abundances of Firmicutes, Bacteroidetes, Verrucomicrobia, and Actinobacteria were observed among the mice generation groups (*p* < 0.05, ANOVA) (Supplementary Table S2). Microbiota composition differences between G1 and G2 mice were characterized by a significant decrease in the relative abundance of Verrucomicrobia (*p* < 0.001; ANOVA with Bonferroni correction), and a significant increased relative abundance of Firmicutes (*p* = 0.012) and Actinobacteria (*p* = 0.009) (**Figure 3**). Significant differences in the relative abundance of specific taxa also persisted beyond this period. While the increase in Bacteroidetes relative abundance between G1 and G2 was not significant, significant differences were seen between G1 and G3 (*p* < 0.001), and between G1 and G6 (*p* = 0.002).

Inter-generational differences were also observed between G3 and G6 mice, with a decrease in the median relative abundances of Verrucomicrobia and Actinobacteria [*p* = 0.039 and *p* = 0.001, respectively, ANOVA (litter nested within generation) with Bonferroni correction]. These changes suggest an ongoing intergenerational variance as opposed to a unidirectional process of acclimation.

**FIGURE 4:**
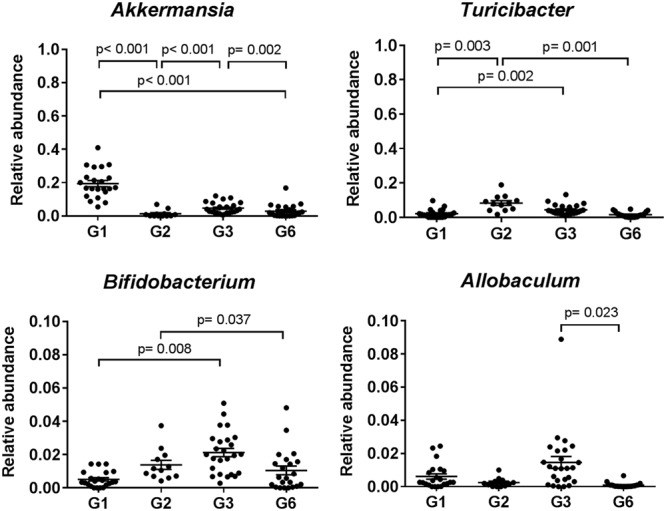
**Taxa specific changes across C57BL/6J mice generations.** Relative abundance (proportion) of selected bacterial genera in the fecal microbiota of C57BL/6J inbred mice that were significantly altered between mice generations. The middle bar and error bars represent the mean and standard error of mean, respectively. Statistical analyses for each bacterial taxon were performed using ANOVA with Bonferroni correction for comparison between generations at a significance level of *p* < 0.05.

SIMPER analysis was used to identify the bacterial taxa that contributed most to the fecal microbiota differences between G1 and G2, as well as subsequent inter-generational differences (determined based on contribution of 70% to the cumulative variance) (Supplementary Figure S4). Significant increases in the mean relative abundances of *Lactobacillus, Clostridium*, and Ruminococcaceae were observed between G1 and G2 mice (*p* < 0.05, ANOVA with Bonferroni correction; Supplementary Figure S4). The mean relative abundance of *Turicibacter* and *Bifidobacterium* also increased between G1, and G2 or G3, respectively (*p* = 0.002 and *p* = 0.008), but decreased at G6 (*p* = 0.001 and *p* = 0.037, respectively) (**Figure 4**). Temporal changes in the relative abundance of *Akkermansia* (primarily accounted for the changes observed for Verrucomicrobia) were observed across the G1, G2, G3, and G6 generations (*p* < 0.05).

### Fecal Microbiota Variation between Mice Generations and within Individuals Over Time

**FIGURE 5:**
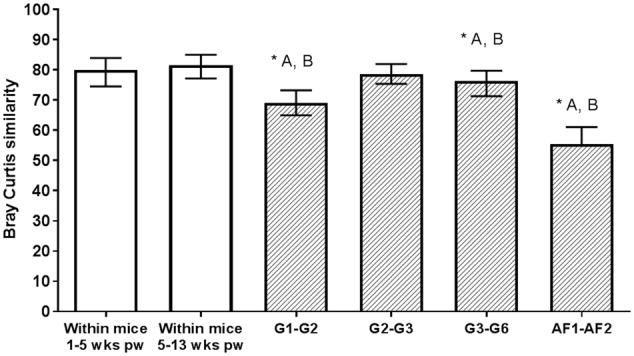
**Microbial community variance between and within individuals.** Within-individual variation of mice fecal microbiota between ages of 1–5 weeks post-weaning (PW) and 5–13 weeks PW (clear bars), and variation between mice generations (shaded bars) measured using Bray–Curtis similarity. Bray–Curtis similarity scores of between mice generations were compared against the within-individual groups, with significant differences to the 1–5 weeks PW group (^∗^A) or 5–13 weeks PW group (^∗^B) denoted above the bars. The bar plots and error bars represent the median and interquartile ranges of the groups. Statistical analyses between groups were performed using the Dunn’s test for multiple comparison correction at a significance level of *p* < 0.05.

Mice in this study ranged from 6 to 8 weeks of age, beyond the point where the murine intestinal microbiome is thought to have reached a mature and stable state (Schloss et al., 2012). Therefore, to determine whether differences in the ages of individual mice might contribute to the inter-generational variance observed, we compared microbiota composition of mice between 1 and 5 weeks PW (4 and 8 weeks of age, respectively), and between mice at 5 and 13 weeks PW (8 and 16 weeks of age, respectively) (**Figure 5**). Larger variations were observed between mice generations, except for variance between G2 and G3, when compared to the within-individual variance over time (*p* < 0.05, Kruskal–Wallis with Dunn’s multiple comparison test). In addition, no significant within-individual differences was observed across all time points (PERMANOVA with Bonferroni correction: *p* = 0.288, 9915 permutations and *p* = 0.577, 9956 permutations, respectively) (Supplementary Table S3).

### Potential Contributors to Compositional Variance

The influence of litter membership on the observed inter-generational differences was assessed by PERMANOVA tests with litter nested within generation. While litter accounted for a greater proportion of variation than generation (Pseudo-*F*: litter = 2.01, generation = 2.79; square root ECV: litter = 7.22, generation = 6.85, residual = 14.34), significant differences were still observed between generation (*p* < 0.0016, 9924 permutations), as also indicated by pairwise analysis between G2 and G3 (*t* = 1.748, *p* = 0.013, 9678 permutations) and G3 and G6 (*t* = 1.693, *p* = 0.030, 5193 permutations) (Supplementary Table S4). The influence of sex of mice in microbiota differences was also assessed by PERMANOVA tests with generation and sex as crossed factors. Although the differences in the fecal microbiota of male and female mice were statistically significant when analyzed across all samples in the inter-generational study as a whole (PERMANOVA *p* = 0.002, 9926 permutations), sex was not a significant influence to the observed variation between mice generations (PERMANOVA *p* = 0.3164, 9866 permutations; Pseudo-*F*: sex = 3.32, gen × sex = 1.10; square root ECV: sex = 4.03, gen × sex = 1.63, residual = 15.70) (Supplementary Table S5).

### Intergenerational Changes in the Fecal Metabolome

**FIGURE 6:**
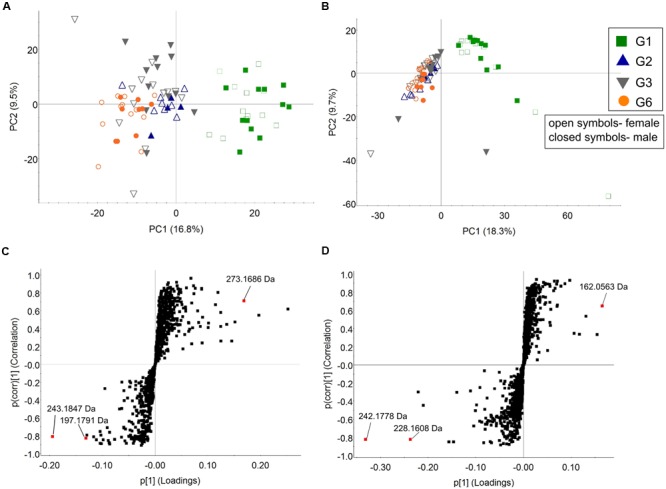
**Fecal metabolome analysis among C57BL/6J mice generations.** Unsupervised PCA analysis of fecal samples of G1, G2, G3, and G6 mice based on **(A)** positive and **(B)** negative ESI markers. The open and closed symbols represent female and male mice, respectively. S-plots were generated from OPLS-DA analysis of **(C)** positive and **(D)** negative markers, with the discriminant markers between the groups as labeled (Model fit: *R*^2^Y = 0.95, *Q*^2^ = 0.93 for positive ionization mode and *R*^2^Y = 0.93, *Q*^2^ = 0.89 for negative ionization mode).

Differences in metabolome composition between the founder populations and subsequent generations assessed by unsupervised PCA indicated that the inter-generational differences in the fecal metabolome mirrored those in microbiota composition (*r* = 0.569, *p* = 0.0001 and *r* = 0.583, *p* = 0.0001, respectively; (**Figures 6A,B**). Metabolite distribution was substantially different between G1 and G2, with significant differences in 237 positive electrospray ionization mode (ESI) markers and 453 negative ESI markers (five-fold change difference in abundance, ANOVA *p* < 0.005). Less marked, but still significant, differences were observed between mice in subsequent generations (G2–G3: 49 positive ESI and 74 negative ESI; G3–G6: 50 positive ESI and 79 negative ESI).

S-plots generated from OPLS-DA analysis was used to identify potential markers that contribute to the difference between two groups, based on their contribution to the model. S-plots from pairwise comparisons between G1 and G6 (**Figures 6C,D**) revealed a number of metabolites that were significantly different in the two populations (Supplementary Table S6). One of the markers, 3-methyldioxyindole, which is potentially derived from tryptophan through the oxidation of 3-methylindole, was significantly higher in the G6 metabolome, compared to G1.

### Assessing the Scale of Observed Microbiome-level Change

To provide an indication of the magnitude of changes in the intestinal microbiome following the introduction of a mouse population to a facility, we compared inter-generational differences between our study population to an outgroup population. We deliberately selected a second population where there were differences in factors known to strongly influence the intestinal microbiome characteristics, including diet, husbandry and barrier conditions (Friswell et al., 2010; Campbell et al., 2012; Rausch et al., 2016). This comparison group, referred to as AF2, were held at a separate facility, where less stringent barrier practices were employed and chow from a different manufacturer was provided. Further, while of the same strain as the first population (C57BL/6J substrain), this second group were derived from a different supplier, and had been held at the facility for more than six generations.

As expected, fecal microbiota composition in AF2 differed significantly to that in the first population (G6, referred to as AF1) (PERMANOVA *p* < 0.0001, 9939 permutations; Pseudo-*F* = 40.91; square root ECV = 25.6, residual = 18.78) (Supplementary Table S7) and clustered separately in the NMDS plot (**Figure 2**). Comparison of alpha diversity measures between the two mice populations also indicated that the AF2 group had significantly higher levels of bacterial richness (*p* < 0.0001, Mann–Whitney test), diversity (*p* < 0.0001) and evenness (*p* < 0.0001) compared to AF1 (Supplementary Figure S5). Although the microbiota of the AF1 and AF2 mice populations differed significantly, no differences were observed in the dispersion of microbial community within each group (Supplementary Figure S5). The magnitude of inter-group differences ranged from those within a single population sampled across an 8 weeks interval at the low end (Bray–Curtis similarity score: 81.62, IQR: 77.16, 85.02), to those between AF1 and AF2 mice and at the high end (Bray–Curtis similarity score: 56.43, IQR: 52.85, 59.23). The divergence observed between G3 and G6 mice (76.41, IQR: 71.33, 79.75), and between G1 and G2 mice (69.06, IQR: 64.95, 73.26) sat between these two extremes (**Figure 5**).

**FIGURE 7:**
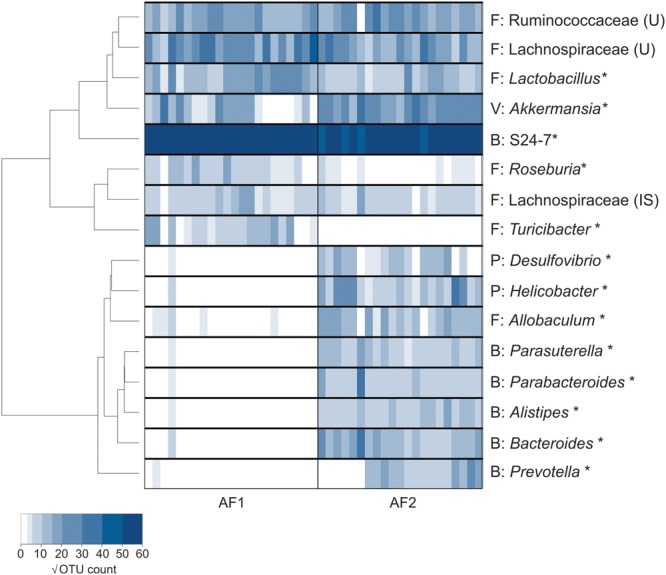
**Microbiota comparison of C57BL/6J mice between two animal facilities.** Heatmap analysis of selected bacterial genera that contributed to the variation (up to 85% of variance) between the two different animal facilities. Bacterial taxa that were significantly different between the groups were denoted with an asterisk (^∗^), as determined by the Mann–Whitney test. Taxa abundances were tabulated as square root-transformed OTU counts. The phylum category for each genera are stated as abbreviations: Bacteroidetes (B), Firmicutes (F), Actinobacteria (A), and Proteobacteria (P).

Inter-group differences were evident in levels of distinct bacterial taxa; AF2 mice lacked *Turicibacter, Bifidobacterium* (uncultured) and *Roseburia* that were found in the fecal microbiota of AF1 mice, while *Desulfovibrio, Prevotella, arasuterella, Parabacteroides, Bacteroides*, and *Helicobacter* were present only in AF2 mice (**Figure 7**). A number of taxa which varied significantly between generations, also contributed substantially to these inter-group differences, including *Turicibacter* (G1 to G2 = 3.98-fold increase; G2 to G6 = 5.26-fold decrease; G6 and AF2 = 342.47-fold lower in AF2), *Bifidobacterium* (G1 to G2 = 2.70-fold increase; G2 to G6 = 1.32-fold decrease; AF1 and AF2 = 104.71-fold lower in AF2), and *Roseburia* (9.95-fold increase; 1.05-fold decrease; 8.62-fold lower in AF2).

**FIGURE 8:**
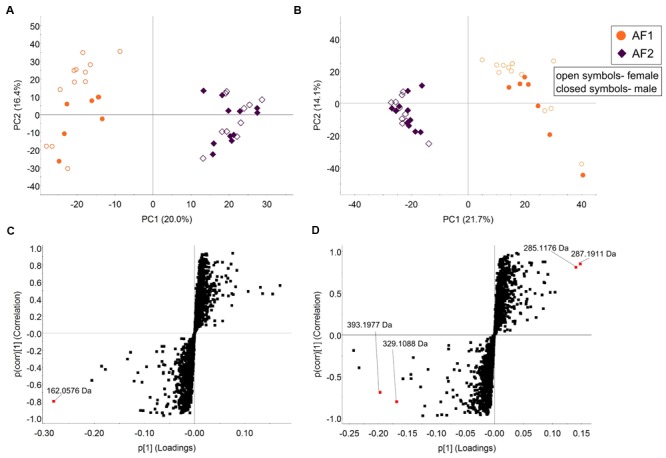
**Fecal metabolome analysis of C57BL/6J mice between two animal facilities.** Unsupervised PCA analysis of fecal samples of C57BL/6J mice from two different animal facilities in the **(A)** positive- and **(B)** negative ion mode. The open and closed symbols represent female and male mice, respectively. S-plots were generated from OPLS-DA analysis of **(C)** positive and **(D)** negative markers, with the discriminant markers between the groups as labeled (Model fit: *R*^2^Y = 0.91, *Q*^2^ = 0.66 for positive ionization mode and *R*^2^Y = 0.96, *Q*^2^ = 0.83 for negative ionization mode).

In keeping with microbiota-level analysis, unsupervised PCA based on AF1 and AF2 metabolome data indicated substantial between-group differences, with significant differences in 684 positive ESI and 850 negative ESI (**Figures 8A,B**, respectively; five-fold change difference in abundance, ANOVA *p* < 0.005). Accordingly, the proportion of positive and negative markers shared between AF1 and AF2 (85.7%), were lower compared to those shared between the mice generation groups (G1–G2: 87.8%; G2–G6: 93.8%). S-plots of OPLS-DA analysis from pairwise comparisons performed between AF1 and AF2 groups (**Figures 8C,D** and Supplementary Table S8) revealed several positive and negative ESI markers that were highly discriminant between the groups (*p* < 0.005, Student’s *t-*test). Biomarkers that significantly differed between the inter-facility group include potential tryptophan metabolites (2-indolecarboxylic acid or 4,6-dihydroxyquinolone) and amino acids, suggesting functional difference in mice between different facilities.

## Discussion

Given the fundamental role of the gut microbiome in many aspects of host physiology, the growing number of reports that implicate microbiota-level differences in the outcomes of *in vivo* experiments are perhaps unsurprising. Variance in the gut microbiome clearly has the potential to affect our ability to replicate experimental findings, and is an essential consideration when designing investigations. Our findings extend previous reports that describe intestinal microbiota changes that occur when mice move from suppliers to research facilities (Fushuku and Fukuda, 2008b; Hoy et al., 2015). We observed substantial changes in both microbiota composition and its functionality, over subsequent mice generations within a highly controlled facility.

The observed differences between the introduction population and second generation animals is on a scale that approached those seen between populations that were subject to differences in major determinants of the intestinal microbiome, including diet, husbandry, and genetic background. Overall, an ongoing increase in the number of bacterial taxa detectable in fecal samples, a reduction in the evenness of their distribution, and variation in the microbiota composition, was observed. Further, there was more subtle but ongoing inter-generational variation in subsequent generations.

Significant genus-level changes were identified between the founder populations and subsequent generations. While the physiological impact of these changes is not known, some of the taxa that contributed substantially to variance are known to influence regulation of host systems considerably. For example, the relative abundance of the *Akkermansia* genus decreased substantially between G1 and G2. *Akkermansia* is known to be involved in mucin-degradation, acetate and propionate production, and modulates the expression of host metabolic genes (Lukovac et al., 2014; Shin et al., 2014; Anhe et al., 2015). *Akkermansia* also appears to be an important regulator of adipose tissue homeostasis, with levels strongly correlated to the lipid metabolism and markers of inflammation (Dao et al., 2014; Schneeberger et al., 2015). Other taxa whose relative abundance changes markedly between G1 and subsequent generations included *Roseburia* and *Clostridium*, which are members of the *Clostridium XIVa* cluster, a group of mucosa-associated SCFA-producing species that has been implicated in immune homeostasis and the amelioration of colonic inflammation in inflammatory bowel diseases (Morgan et al., 2012; Machiels et al., 2014). Taxa in the *Clostridium XIVa* cluster have been shown to induce the production of the anti-inflammatory cytokine IL-10 by T regulatory cells and inhibit the proinflammatory NF-κB pathway, implicated in tumor cell apoptosis (Hague et al., 1995; Atarashi et al., 2011; Lopetuso et al., 2013). Ongoing instability in the relative abundances of *Turicibacter* and *Bifidobacterium* was observed across the generations assessed. *Turicibacter* levels have been linked to immune phenotypes, including the population levels of marginal zone B cells and invariant natural killer T cells (Presley et al., 2010). Temporal changes in the relative abundances of *Bifidobacterium*, a genus associated with immunomodulation, and the basis of some probiotic treatments (Toumi et al., 2014), were also observed.

The rate and nature of change of the microbiota following introduction of mice into a facility will be influenced by factors such as the extent of differences between the two sites, as well as a range of external selective factors (Rausch et al., 2016). This process of acclimation will depend on the stochastic loss or gain of taxa, combined with the effect of husbandry conditions such as chow treatment and the potential for contact with novel taxa (for example, the implementation of barrier conditions). The vertical transmission of intestinal microbiota during birth and nursing, as demonstrated by cross-fostering and embryo-implantation experiments (Daft et al., 2015), is likely to further contribute to this process and has been shown to be particularly susceptible to stochastic change (Rausch et al., 2016; Sonnenburg et al., 2016). However, other potential contributors must be considered. For example, the composition of the intestinal microbiota changes substantially during mouse development, and between group differences could therefore lead to generational variance. However, we assessed mice of an age substantially beyond that where the murine intestinal microbiota is thought to be mature and stable (11–17 days PW) (Schloss et al., 2012). Furthermore, our assessment of within-individual variation indicated that the gut microbiota composition of mice did not significantly differ at 4, 8, and 16 weeks old, suggesting that the changes between generations were due to other factors than weaning. Differences in sex distribution might also represent a potential bias, despite having been shown previously to be associated with only a low level of microbiota variation in mice (Fushuku and Fukuda, 2008a; Campbell et al., 2012), of which some might be due to cage effects from the separate housing of sexes (Friswell et al., 2010). The failure to observe any sex-related variance here further suggests that sex distribution cannot explain inter-generational variance. While the influence of cage effects could not be measured within our intergenerational assessments, the distribution of mice did not differ between the generational groups assessed. Further, C57BL/6J mice strains have been previously shown to be less susceptible to co-caging effects compared to other mice strains (Deloris Alexander et al., 2006; Campbell et al., 2012). The influence of litter membership was also assessed. While litter membership did account for a substantial proportion of the microbiome variation observed, it did not fully account for the differences between generations.

The gut microbiome is believed to contain substantial functional redundancy, with multiple bacterial taxa capable of contributing to similar metabolic outcomes (Moya and Ferrer, 2016). Differences in microbiota composition do not, therefore, necessarily result in alterations to the overall functional output of the gut microbiome. However, we observed inter-generational differences in microbiota composition to be associated with corresponding differences in the fecal metabolome, consistent with previous studies indicating a close correlation between microbiota metabolome composition (Larsen and Dai, 2015). Indeed, it has been demonstrated that metabolome characteristics provide a better indication of intestinal dysbiosis than microbiota composition (Larsen and Dai, 2015). The physiological impact of discriminant metabolites, such as the inter-group variation in tryptophan pathway metabolites and amino acids, was not assessed as part of this study. However, these pathways have previously been shown to have potential influence on processes such as immune regulation, colonization resistance, metabolic homeostasis, and central nervous system function (Bernstein et al., 2011; Vinolo et al., 2011; Theriot et al., 2014), and warrant further investigation.

We assessed mice held at separate facilities to provide a comparison for levels of inter-generational variance. A second population was deliberately selected that differed in factors that are known to strongly influence the intestinal microbiome characteristics, including diet, husbandry, and genetic background. In keeping with previous reports, substantial inter-group differences were observed (Ericsson et al., 2015). These included substantial differences in taxa that are likely to significantly influence host physiology, including *Desulfovibrio, Bacteroides, Parabacteroides, Prevotella*, and *Allobaculum*. The changes in these taxa can, for example, render the mucus layer more penetrable (Jakobsson et al., 2015), a phenomenon that is implicated in the development of spontaneous colitis (Hale and Greer, 2012). Again, differences in microbiota composition were reflected in changes in the metabolome, including levels of 2-indolecarboxylic acid and 4,6-dihydroxyquinolone, which form part of the tryptophan pathway and are involved in immune modulation, inflammation and affects intestinal function (Zheng et al., 2011; Zhang et al., 2012). Levels of amino acids, such as glutamine, glutamate and aspartate, which are important in intestinal epithelial cell layer renewal and nutrient absorption (Blachier et al., 2009), and also act as a precursor for microbial production of SCFAs (Neis et al., 2015), also differed significantly.

This study had a number of limitations that should be considered. First, 16S rRNA gene amplicon sequencing and LC-MS profiling for characterization of microbiota composition and the fecal metabolome, respectively, are imperfect. For example, 16S-based approaches can be influenced by differences in ribosomal operon copy number between taxa (Kembel et al., 2012), while LC-MS output does not always allow confident identification of all detected metabolites (Xiao et al., 2012). Further, the number of mice that were assessed will contribute to the relative impact of factors such as cage and litter effects on inter-generational changes. The future application of the increasingly sophisticated microbiome characterization strategies to larger mouse populations will further refine the observations reported here.

## Conclusion

By assessing changes that occur following the introduction of a mouse strain into a research facility, our study models a process that has taken place in the majority of animal populations used in research. We observed significant changes in the microbiota, particularly between the founder population and the second generation, but also between generations of mice kept in the same facility. The reflection of these differences in the metabolome suggests the potential to alter a range of host metabolism pathways that are investigated as experimental outcomes, or affect the reproducibility of studies. Controlling for differences in microbiota composition between experimental populations is challenging. However, by identifying both a highly dynamic process of acclimation that follows population introduction into a facility, and an ongoing variance in the characteristics of the intestinal microbiome, this study highlights an important consideration for the design of experiments involving mice.

## Author Contributions

JC and PT contributed to the execution, design, analysis and writing of the manuscript. CB and NJ contributed to the execution of the study, LL, GA, TD, SW, MS, and GR contributed to the analysis and writing of the manuscript. All authors read and approved the final manuscript.

## Conflict of Interest Statement

The authors declare that the research was conducted in the absence of any commercial or financial relationships that could be construed as a potential conflict of interest.
