# Supplementary material for: Inbred Mouse Populations Exhibit Intergenerational Changes in Intestinal Microbiota Composition and Function Following Introduction to a Facility
Source: Front Microbiol. 2017 Apr 11;8:608. doi: 10.3389/fmicb.2017.00608 (PMC5387074; doi:10.3389/fmicb.2017.00608)
Supplement: Supplementary file 1 [file Data_Sheet_1.pdf]

Supplementary Material

Inbred mouse populations exhibit substantial intergenerational changes in intestinal microbiota composition and function following introduction to a facility

Jocelyn M Choo[1], Paul J Trim[2], Lex EX Leong[1], Guy CJ Abell[4], Carly Brune[3], Nicole Jeffries[3], Steve Wesselingh[1], T Neil Dear[1], Marten F Snel[2], Geraint B Rogers[1,4*]

[1] Infection and Immunity Theme, South Australia Health and Medical Research Institute, North Terrace, Adelaide, SA, Australia.

[2] LDRU, Nutrition and Metabolism Theme, South Australia Health and Medical Research Institute, North Terrace, Adelaide, SA, Australia.

[3] Bioresources facility, South Australia Health and Medical Research Institute, North Terrace, Adelaide, SA, Australia.

[4] School of Medicine, Flinders University, Bedford Park, Adelaide, SA, Australia.

*** Correspondence:**

Associate Professor Geraint Rogers

School of Medicine, Flinders University

University Drive, Bedford Park,

Adelaide SA 5042, Australia

geraint.rogers@sahmri.com

Tel: +61 (0)8 8204 7614

SUPPLEMENTARY MATERIAL

Supplementary Figures and Tables

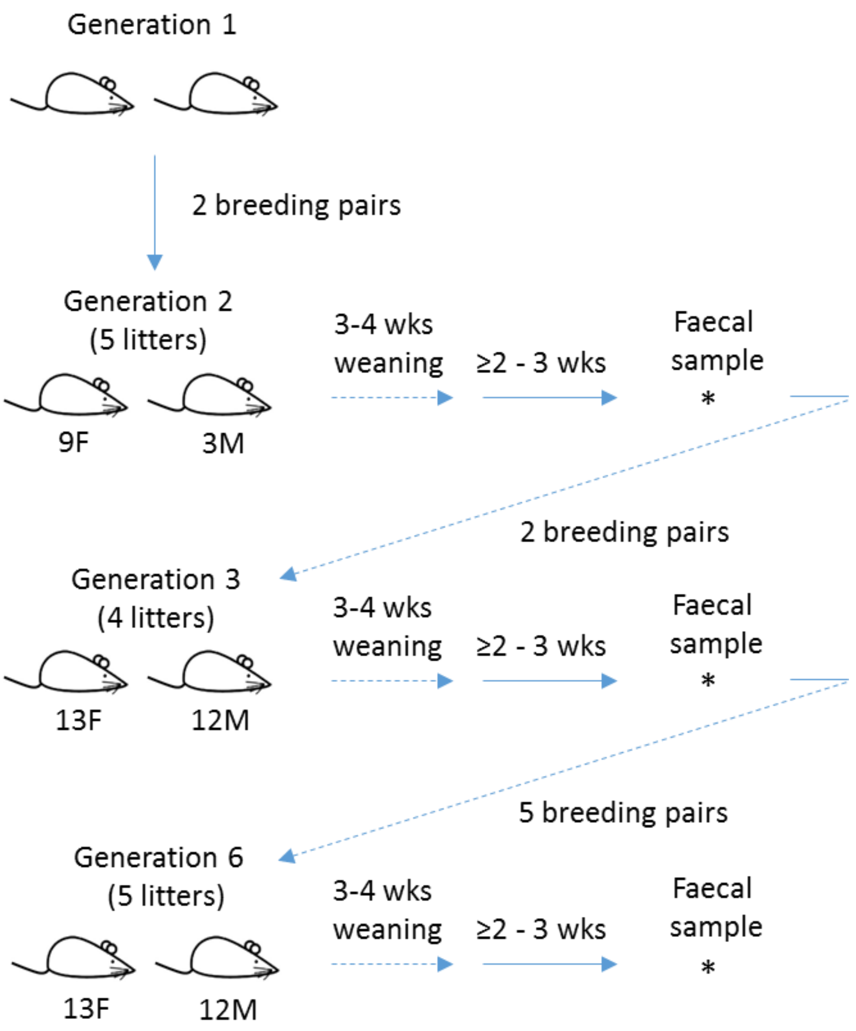

Figure S1. Breeding scheme of C57BL/6J inbred mice generations from G1 to G6.

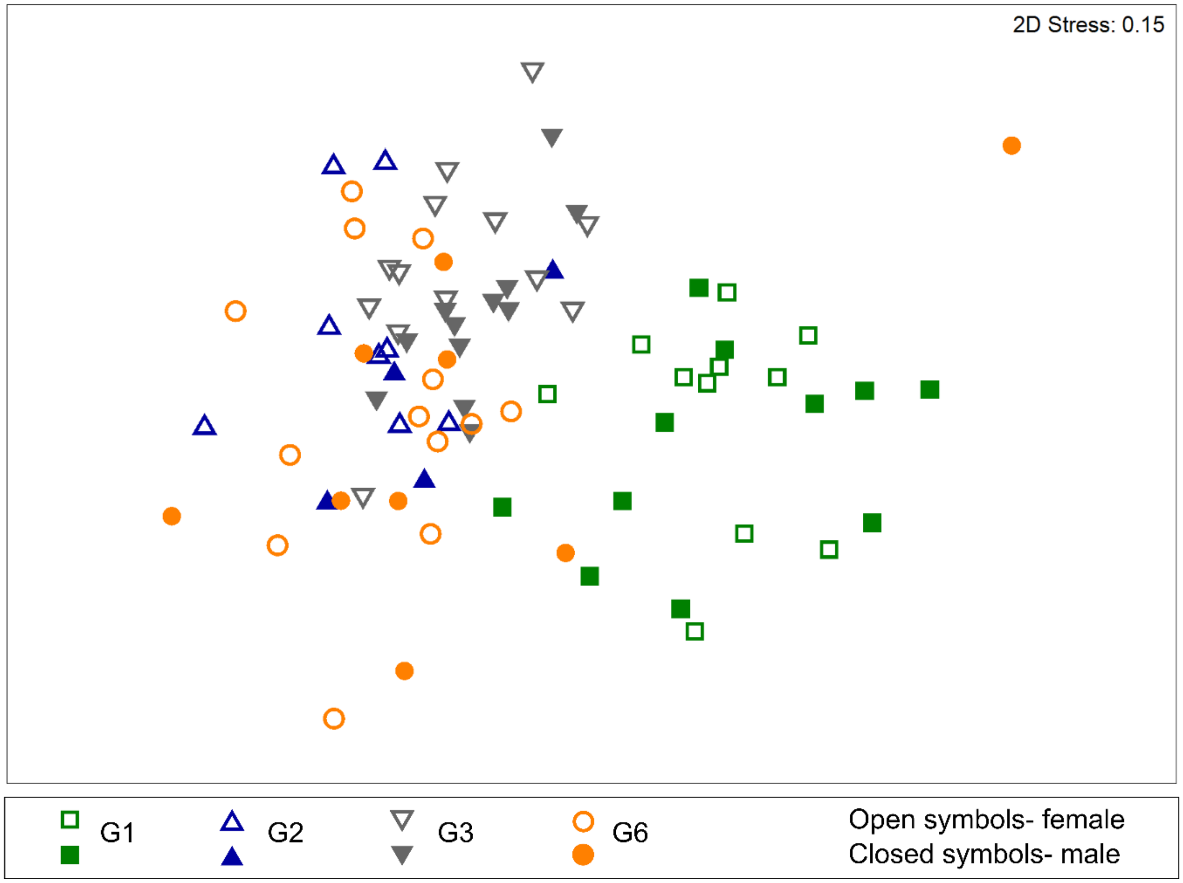

Figure S2. Non-metric multidimensional scaling (NMDS) ordination plot based on the Bray Curtis distances of the faecal microbiota of C57BL/6J inbred mice from the founder (G1), second (G2), third (G3) and sixth (G6) generation within a single facility. Mice from the different generations and facility are labelled as indicated. The open and closed symbols represented female and male mice, respectively.

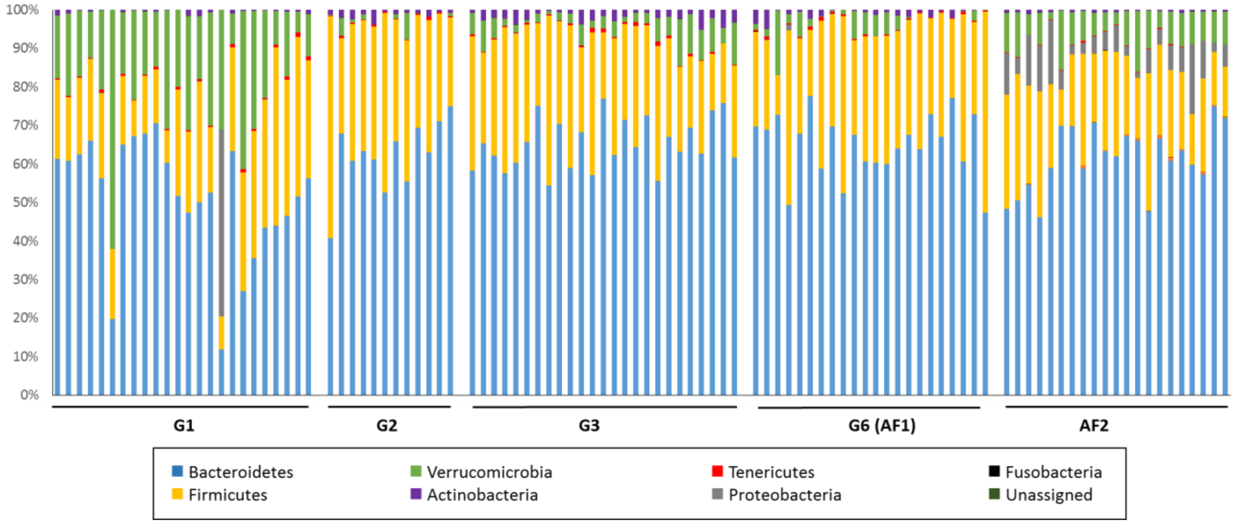

Figure S3. Phylum level relative abundances of the faecal microbial community of G1, G2, G3 and G6 mice populations of the C57BL/6J inbred strain from a single facility, and C57BL6J inbred mice from a different facility (AF2).

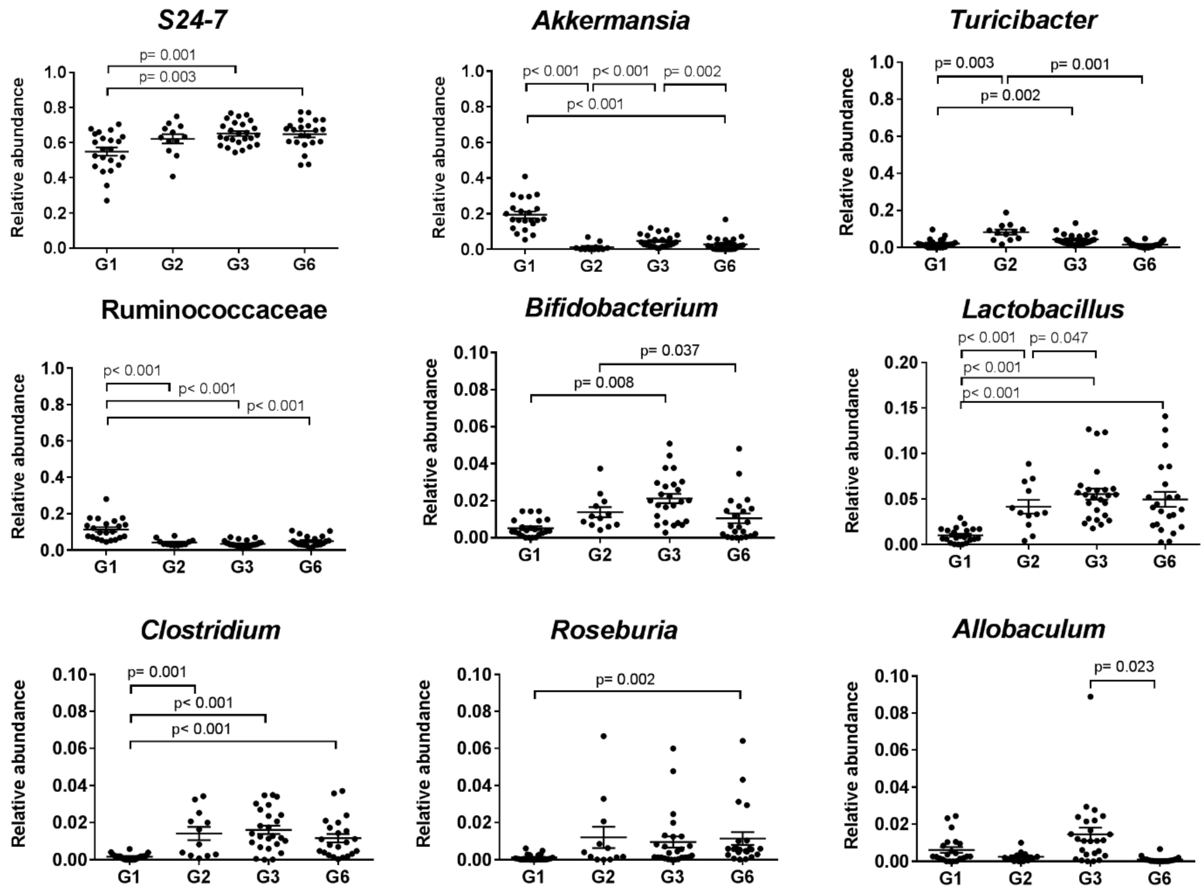

Figure S4. Relative abundances (proportion) of bacterial taxa that contribute (up to 70% cumulative variation) to the differences observed among the mice generations based on SIMPER analysis. Statistical analyses for each bacterial taxa was performed using ANOVA (litters nested within generation) with Bonferroni correction for multiple comparison between mice generations.

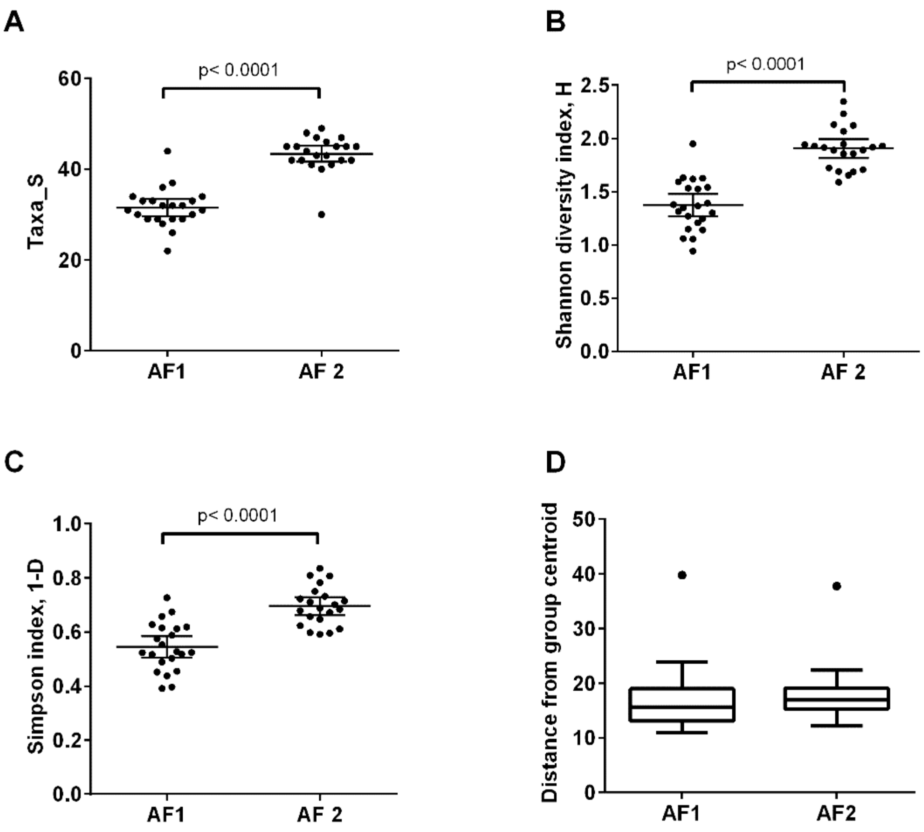

Figure S5. Alpha diversity analysis of C57BL/6J inbred mice faecal microbiota from two different facilities, AF1 and AF2, based on (A) microbial richness, (B) Shannon-Wiener index *H* for microbial diversity and (C) Simpson index (1-D) for microbial evenness, which takes into account microbial richness. (D) The dipersion of microbial community within the two mice populations were also assessed using PERMDISP analysis. Statistical analysis between the groups were performed using the Mann-Whitney test at a significance level of $P < 0.05$.

Table S1. PERMANOVA analysis on Bray Curtis distances of the faecal microbiota of inbred mice over four different mice generations.

Source	df	SS	MS	Pseudo-*F*	Square root ECV	Permutations	*P*
Generation	3	9703.4	3234.5	12.65	12.25	9916	0.0001
Residual	73	19675	246.4		15.99		
Total	80	29378					

Comparison	*t*	Permutations	*P*	*P**
G1 versus G2	4.2255	9939	0.0001	0.0006
G1 versus G3	4.7569	9941	0.0001	0.0006
G1 versus G6	4.6326	9931	0.0001	0.0006
G2 versus G3	2.2369	9919	0.0002	0.0012
G2 versus G6	2.0054	9930	0.0005	0.0030
G3 versus G6	2.9451	9942	0.0001	0.0006

df = degree of freedom, SS= sum of squares, MS = mean sum of squares, ECV= estimates of components of variation, *t* = test statistic, Pseudo-*F*= *F* value based on permutation test,
P= PERMANOVA *P*-value, P*= Bonferroni-adjusted *P*-value.

Table S2. Median percentage relative abundance of bacterial taxa at the phylum level, of C57BL/6J inbred mice from four different generations and from a different animal institution.

**Phyla**	**Median relative abundances (%)**				
	**G1**	**G2**	**G3**	**G6 (AF1)**	**AF2**
Actinobacteria *[a]	0.45	1.15	2.11	0.63	0.53
Bacteroidetes [a]	56.32	63.15	64.28	67.26	62.01
TM7	0	0	0	0	0.19
Cyanobacteria	0	0	0	0	0
Deferribacteres	0	0	0	0	0
Firmicutes [a]	21.66	34.22	25.60	30.20	22.54
Fusobacteria	0	0	0	0	0
Proteobacteria	0.02	0	0	0.033	5.59
Tenericutes	0.49	0.33	0.44	0.33	0.13
Verrucomicrobia *[a]	16.87	0.47	4.03	1.78	8.32
Unassigned	0.016	0.016	0.016	0	0.03
Firmicutes/ Bacteroidetes	0.41	0.54	0.38	0.46	0.37

* phylum abundance significantly different across mice generation groups G2, G3 and G6 ($P < 0.05$, ANOVA (litter nested within generation) with Bonferroni's correction)

[a] phylum abundance significantly different between G1 and one or more of the subsequent mice generation G2, G3 and G6 ($P < 0.05$, ANOVA with Bonferroni's correction)

Table S3. Permutational multivariate analysis of variance (PERMANOVA) analysis, based on time and mice as crossed factors, on Bray Curtis distances of the microbial community of C57BL/6J inbred mice faecal samples collected at 1 week, 5 weeks and 13 weeks post-weaning to assess within-individual variation.

Source	df	SS	MS	Pseudo-*F*	Square root ECV	Permutations	*P*
Time	2	751.36	375.68	1.89	4.37	9927	0.1456
Mice	7	1698.40	242.62		1.87	9926	0.4416
Residual	22	5031.2	228.7		15.12		
Total	24	5893.9					
Pairwise analysis				*t*	Permutations	*P*	*P**
1 wk pw versus 5 wks pw				1.398	9915	0.1442	0.2884
5 wk pw versus 13 wks pw				1.145	9956	0.2883	0.5766

df = degree of freedom, SS= sum of squares, MS = mean sum of squares, ECV= estimates of components of variation, Pseudo-*F*= F value based on permutation test, *t* = test statistic, P= PERMANOVA *P*-value, P*= Bonferroni-adjusted *P*-value, pw= post-weaning.

Table S4. PERMANOVA analysis on Bray Curtis distances of the faecal microbial composition over four mice generations with litter groups included as a factor.

Source	df	SS	MS	Pseudo-*F*	Square root ECV	Permutations	*P*
Generation	2	2173.3	1086.6	2.79	6.85	9924	0.0016
Litter (Gen)	10	4140.0	414.0	2.01	7.22	9836	0.0005
Residual	41	8433.4	205.7		14.34		
Total	53	15481					

Comparison	*t*	Permutations	*P*	*P**
G2 versus G3	1.748	9678	0.0043	0.0129
G2 versus G6	1.456	8497	0.0517	0.1551
G3 versus G6	1.693	5193	0.0098	0.0294

df = degree of freedom, SS= sum of squares, MS = mean sum of squares, ECV= estimates of components of variation, Pseudo-*F*= F value based on permutation test, *t* = test statistic, P= PERMANOVA *P*-value, P*= Bonferroni-adjusted *P*-value.

Table S5. PERMANOVA analysis on Bray Curtis distances of the faecal microbial composition of C57BL/6J mice over four different generations with sex of mice included as a factor.

Source	df	SS	MS	Pseudo-*F*	Square root ECV	Permutations	*P*
Generation	3	9229.5	3076.5	12.48	12.13	9901	0.0001
Sex	1	819.25	3.3244	3.32	4.03	9926	0.0024
Gen x Sex	3	815.83	271.94	1.10	1.63	9866	0.3164
Residual	73	17990	246.43		15.70		
Total	80	29378					

Generation	Group	*t*	Permutations	*P*	*P**
G1	F vs M	1.4715	9787	0.0463	0.1852
G2	F vs M	1.0268	494	0.4205	1
G3	F vs M	1.3222	9922	0.0810	0.3240
G6	F vs M	1.3465	9837	0.0629	0.2516

df = degree of freedom, SS= sum of squares, MS = mean sum of squares, ECV= estimates of components of variation, Pseudo-*F*= F value based on permutation test, *t* = test statistic, P= PERMANOVA *P*-value, P*= Bonferroni-adjusted *P*-value.

Table S6. Potential markers identified to be significantly different between G1 and G6 mice based on S-plot analysis.

**Positive markers**				
m/z	**Biomarker mass**	**Adduct**	**Potential biomarker**	**Formula**
180.1758<br><br>(Neutral mass= 197.1791 Da)	197.1780	M	N-methylundec-10-enamide	C12H23NO
	197.1780	M	((5R,8R)-5-propyloctahydroindolizin-8-yl)methanol	C12H23NO
	197.1780	M	(5-ethyl-6-methyloctahydroindolizin-8-yl)methanol	C12H23NO
	197.1780	M	8-methyl-5-propyloctahydroindolizin-8-ol	C12H23NO
226.1814 (Neutral mass= 243.1847 Da)	N/A	N/A	N/A	N/A
273.1686	290.1729	[M+H-H2O]+	1-Octen-3-yl glucoside	C14H26O6
162.0563	163.0633	[M-H]-	3-Methyldioxyindole	C9H9NO2
	163.0633	[M-H]-	4-Oxo-1-(3-pyridyl)-1-butanone	C9H9NO2
	163.0633	[M-H]-	5-Hydroxy-3,4-dihydrocarbostyryl	C9H9NO2
	163.0633	[M-H]-	p-Acetaminobenzaldehyde	C9H9NO2
	163.0633	[M-H]-	4-(3-Pyridyl)-3-butenoic acid	C9H9NO2
228.1608	N/A	N/A	N/A	N/A
242.1778	N/A	N/A	N/A	N/A

Table S7. Permutational multivariate analysis of variance (PERMANOVA) analysis on Bray Curtis distances of the faecal microbiota of G6 and AF2 group, which are C57BL/6J inbred mice from different animal institutions. Pairwise analysis was performed between all generation of mice against the inter-facility group, AF2.

Source	df	SS	MS	Pseudo-*F*	Square root ECV	Permutations	*P*
Facility	1	14430	14430	40.91	25.60	9939	0.0001
Residual	41	14463	352.8		18.78		
Total	42	28893					
Group				*t*	Permutations	*P*	*P**
G1, AF2				6.5802	9936	0.0001	0.0004
G2, AF2				6.3729	9935	0.0001	0.0004
G3, AF2				7.6081	9928	0.0001	0.0004
G6, AF2				6.3959	9922	0.0001	0.0004

df = degree of freedom, SS= sum of squares, MS = mean sum of squares, Pseudo-*F*= F value based on permutation test, ECV= estimates of components of variation, *t* = test statistic, P= PERMANOVA *P*-value, P*= Bonferroni-adjusted *P*-value.

Table S8. Potential markers identified to be significantly different between inter-facility mice groups (G6 and AF2) based on S-plot analysis.

**Positive markers**				
m/z	**Biomarker mass**	**Adduct**	**Potential biomarker**	**Formula**
162.0576<br><br>(Neutral mass= 161.0487 Da)	161.0477	M	2-Indolecarboxylic acid	C9H7NO2
	161.0477	M	Indole-3-carboxylic acid	C9H7NO2
	161.0477	M	4,6-Dihydroxyquinoline	C9H7NO2
	161.0477	M	4,8-Dihydroxyquinoline	C9H7NO2
	161.0477	M	Quinoline-3,4-diol	C9H7NO2
	161.0477	M	Quinolin-2,8-diol	C9H7NO2
	161.0477	M	3-Hydroxy-1H-quinolin-4-one	C9H7NO2
	161.0477	M	4-Hydroxy-2-quinolone	C9H7NO2
	161.0477	M	3-Formyl-6-hydroxyindole	C9H7NO2
287.1911	N/A	N/A	N/A	N/A
393.1977	N/A	N/A	N/A	N/A
**Negative markers**				
285.1176	286.1230	[M-H]-	Zolazepam	C15H15FN4O
	286.1277	[M-H]-	Gly Gly Gly Pro	C11H18N4O5
	286.1277	[M-H]-	Gly Gly Pro Gly	C11H18N4O5
	286.1277	[M-H]-	Gly Pro Gly Gly	C11H18N4O5
	286.1277	[M-H]-	Pro Gly Gly Gly	C11H18N4O5

	286.1277	[M-H]-	Asn Gly Pro	C11H18N4O5
	286.1277	[M-H]-	Pro Asn Gly	C11H18N4O5
	286.1277	[M-H]-	Gly Pro Asn	C11H18N4O5
	286.1277	[M-H]-	Gly Asn Pro	C11H18N4O5
	286.1277	[M-H]-	Asn Pro Gly	C11H18N4O5
	286.1277	[M-H]-	Pro Gly Asn	C11H18N4O5
329.1088	330.1135	[M-H]-	Halofenozide	C18H19ClN2O2

Supplementary Materials and Methods

Sample collection, DNA extraction, and 16S rRNA gene amplicon sequencing

Faecal samples were collected by placing individual mice in a clean cage. Fresh faecal pellets were transferred using a sterile toothpick to a 1.5 mL Eppendorf tube and stored at -80°C prior to analysis. Faecal pellets were dispersed in 1mL of phosphate buffered saline (PBS, pH 7.2) by vortexing, and pelleted by centrifugation at 13 000 x g for 5 min. Supernatant was transferred to a sterile 2 mL screwcap tube and stored at -80°C for liquid chromatography mass spectrometry (LC-MS) analysis, while pellets underwent DNA extraction using a PowerSoil®-htp 96 Well Soil DNA Isolation kit (MoBio Laboratories, Carlsbad, USA) with several modifications. Faecal samples were resuspended in 750 μL of PowerSoil® bead solution and 60 uL of solution C1 for chemical cell lysis. Modifications to manufacturer's instructions were the inclusion of a 20 min incubation of the suspension at 65°C, prior to mechanical lysis by bead-beating twice using a mixer mill MM400 96-well plate shaker at 20 vibrations/sec for 10 mins each time (Retsch GmbH, Haan, Germany). The beadplate was then centrifuged at 3374 x g for 10 mins at room temperature and the supernatant transferred to a fresh C2 collection plate. Procedures involving solution C2, solution C3 and solution C4 was performed according to the manufacturer's protocol. The supernatant was then transferred onto a spin filter plate and passed through the membrane filter using a vacuum manifold to capture the DNA onto the membrane. DNA bound to the spin filter were washed with 500 uL of solution C5, an ethanol-based solution, according to the protocol. Total DNA was eluted in 100 uL of sterile water and quantified fluorometrically with a Qubit dsDNA HS Assay kit (Life Technologies, Melbourne, Australia).

Amplicons of the V4 hypervariable region of the bacterial 16S rRNA gene was amplified from faecal DNA extracts as described previously (Choo et al., 2015). Modified universal bacterial primer pairs 515F (5'-<u>TCGTCGGCAGCGTCAGATGTGTATAAGAGACAG</u>GTGCCAGCMGCCGCGGTAA-

3') and 806R (5'-GTCTCGTGGGCTCGGAGATGTGTATAAGAGACAGGGACTACHVGGGTWTCTAAT-3'), with Illumina adapter overhang sequences (indicated by underline) were used for the amplification of the V4 hypervariable region of the bacterial 16S rRNA gene. The amplicons were generated from DNA extracts (25 PCR cycles for amplicon generation and 8 PCR cycles for indexing), cleaned and sequenced according to the Illumina MiSeq 16S Metagenomic Sequencing Library Preparation protocol with certain modifications. Specifically, PCR for amplicon generation was performed at a melting temperature of 50°C.

Samples were multiplexed using a dual-index approach with the Nextera XT Index kit (Illumina, San Diego, USA) according to the manufacturer's instructions. Amplicon library concentrations were determined using the Qubit HS dsDNA assay kit (Life Technologies, Melbourne, Australia). The final library was paired-end sequenced at 2 x 300 bp using a MiSeq Reagent Kit v3 on a Illumina MiSeq platform (Illumina, San Diego, USA), at the David R Gunn Genomics Facility, South Australian Health and Medical Research Institute.

LC-MS analysis

Faecal supernatant, derived from 2.5 mg of faecal matter in PBS, was thawed and vortexed. A 50 µL aliquot was placed in a pre-washed (1 mL acetonitrile) and equilibrated (1 mL 0.1% TFA aqueous) Oasis HLB 10 mg SPE cartridge (Waters Corporation, Milford, MA, USA). The sample was washed with 1 mL 0.1% TFA and eluted with 1 mL 0.1% TFA in 70% acetonitrile. The eluent was lyophilized overnight in a RVC 2-33CD plus rotational vacuum concentrator (Martin Christ Gefriertrocknungsanlagen GmbH, Osterode am Harz, Germany) operated at 10 mBar and room temperature. Samples were reconstituted in 50 µL 0.1% FA, vortexed and centrifuged at 16 000 x g

for 15 min. The supernatant was transferred into LC-MS-grade glass vials (avoiding the pellet) and stored at 6°C until use.

LC-MS was performed on a quadrupole orthogonal acceleration time-of-flight mass analyser (SYNAPT HDMS, Waters Corporation, Milford, MA, USA) coupled to a UPLC system (ACQUITY, Waters Corporation, Milford, MA, USA). Reverse-phase chromatographic separation used a Waters BEH C18 column, 1.7 µm particle size, 2.1 mm i.d. x 150 mm, (Milford, MA, USA).

To assess LC-MS system variability throughout an analysis run, a pooled biological quality control (PBQC) was prepared by combining 10 µL from each sample.

Samples were analysed in random order interspersed with PBQC measurements every fourth analysis.

Sample (12.5 µg faecal weight equiv) was loaded onto the analytical column in 98% mobile phase A (0.1% aqueous formic acid *v/v*) and 2% mobile phase B (0.1% formic acid in acetonitrile *v/v*) at a flow rate of 400 µL/min. The loading conditions were maintained for 0.5 min after which a 12 min linear solvent gradient was applied with a final concentration of 2% A. This concentration was held for 4 min to wash the column after which the solvent mix was returned to starting conditions (98% A) for 2.8 min to re-equilibrate the column ready for the next injection. The same gradient was used for both positive and negative ionisation mode experiments.

Two mass spectrometry experiments were performed, one in negative and one in positive ionisation. For both modes the instrument was calibrated over the acquisition *m/z* range of 50 – 1500 prior to analysis and mass accuracy was maintained during data acquisition by infusion of a reference solution through the instrument's lockmass channel (200 fmol/µL leucine enkephalin (Sigma-Aldrich, Castle Hill, NSW, Australia) in 1:1 methanol:0.1% aqueous formic acid *v/v*) which was sampled every 10 s. Source conditions were optimised for each ionisation mode.

Statistical analysis

Microbial data were analysed for alpha diversity measures (taxa richness, *S*; Shannon-Wiener index, *H*; Simpson diversity index, 1-*D*) of microbial community were determined using PAST (v.3.04) (Hammer et al., 2001). Operational taxonomic unit (OTU) relative abundance was imported into the Primer-E software (v.6, PRIMER-E Ltd, Plymouth, UK) for beta diversity analysis. Bray Curtis similarities were calculated based on the square root-transformed OTU relative abundances, and were used in the non-metric multidimensional scaling (NMDS) ordination plot. Permutational analysis of variance (PERMANOVA) model was used for testing the null hypothesis of no difference (Anderson and Walsh, 2013), based on the parameters permutation of residuals under a reduced model and a type III sum of squares. The bacterial taxa that contributed to the dissimilarities between the groups were determined by SIMPER analysis (Clarke, 1993). PERMDISP was used to assess the dispersion of the microbial community within the groups (Anderson and Walsh, 2013). Comparisons between the microbial and metabolome abundance data was performed using the RELATE analysis (Clarke and Warwick, 2001). Differences in the relative abundance of phyla and genera between generation groups were, where possible, tested for statistical significance based on a nested ANOVA analysis (litters nested in generation), using a type III sum of squares approach on log transformed values in SPSS (v22.0). Multiple pairwise comparisons between groups were performed on the estimated marginal means, with Bonferroni correction applied. Comparisons between generation and within-individual variance was performed using the Kruskal-Wallis test with Dunn’s multiple comparison test using GraphPad PRISM 6 (GraphPad Software Inc., La Jolla, USA). Heatmap was generated in R using the *ggplots2* (v2.0.0) package (Team, 2015; Wickham and Chang, 2015). The unassigned taxa comprised of less than 0.1% of the relative abundance and were not included in the phyla relative abundance

comparison. The DNA and metabolome samples of two mice from G1 and one mouse from G6 failed quality control thresholds and were excluded from microbiota and metabolome analysis, respectively, as the number of observed taxa were very low (less than 20) and the sample was too dilute. G1 mice, and five mice of G6, were not included in assessment of litter effects as data for the maternal origins of these mice were not available.

LC-MS-based metabolomics data were processed using the Progenesis QI software (v2.2, Nonlinear Dynamics, Newcastle Upon Tyne, UK) using default settings. All LC-MS datasets were aligned on retention time, and *m/z* scales and peak areas were compared. For positive ion data, adduct ions (H^+, Na^+ and K^+) and neutral loss of water ions were identified automatically based on retention time and mass accuracy and combined. For reporting purposes data were filtered to only include markers based on the parameters of ANOVA p-value ≤ 0.005, and at least a 5-fold change in lowest and highest mean intensity between sample groups. Identified markers were exported to EZinfo (v3.0, Umetrics, Umea, Sweden) for OPLS-DA (Orthogonal Projection to Latent Structure-Discriminant Analysis) to generate the S-plots using centred, pareto-scaled data for the identification of discriminant markers between groups. The total variation explained by model (R^2Y) and predictability of the OPLS-DA model (Q^2) were determined according to EZinfo. Significant markers were searched in the METLIN database for potential biomarkers that were within the 10 ppm range of the *m/z* (or neutral mass if available), and against the [M-H] negative ions or [M+H], [M+Na] and [M+H-H20] positive ions in the METLIN database (Smith et al., 2005). Positive markers with a neutral mass value were only compared against neutral mass searches in METLIN. All statistical analysis were performed at a significance level of 0.05.

References

Anderson, M., and Walsh, D. (2013). PERMANOVA, ANOSIM, and the Mantel test in the face of heterogeneous dispersions: What null hypothesis are you testing? *Ecological Monographs* 83(4)**,** 557-574.

Choo, J.M., Leong, L.E.X., and Rogers, G.B. (2015). Sample storage significantly influence faecal microbiome profiles. *Scientific Reports* 5**,** 16350.

Clarke, K. (1993). Non-parametric multivariate analyses of changes in community structure. *Australian Journal of Ecology* 18(1)**,** 117-143.

Hammer, O., Harper, D.A.T., and Ryan, P.D. (2001). PAST: paleontological statistics software package for education and data analysis. *Palaentologia Electronica* 4**,** 9.

Smith, C.A., O'Maille, G., Want, E.J., Qin, C., Trauger, S.A., Brandon, T.R., et al. (2005). METLIN: a metabolite mass spectral database. *Ther Drug Monit* 27(6)**,** 747-751.

Team, R.C. (2015). R: A language and environment for statistical computing. *R Foundation for Statistical Computing, Vienna, Austria*.

Wickham, H., and Chang, W. (2015). An Implementation of the Grammar of Graphics. *CRAN*.
